# Supplementary material for: Oats Lower Age-Related Systemic Chronic Inflammation (iAge) in Adults at Risk for Cardiovascular Disease
Source: Nutrients. 2022 Oct 25;14(21):4471. doi: 10.3390/nu14214471 (PMC9656573; doi:10.3390/nu14214471)
Supplement: Supplementary file 1 [file nutrients-14-04471-s001.zip › nutrients-1954822-supplementary.pdf]

Table S1. Composition of investigational Oat product compared to the isocaloric placebo rice flower control.

	Oat product (SoluOBC)	Rice Ctrl (PCB)
Weight (g)	23	22
Energy (kcal)	83	81
Total fat (g)	1	1
Saturated fat (g)	0	0
Total carbohydrates (g)	17	18
Available carbohydrates (g)	14	16
Total fiber (g)	2	0.3
Beta-Glucan (g)	1	0
Avenanthramides (mg)	0.38	0
Total Phenolics (mg)	15.6	11.4
GABA (mg)	0.607	0.368
Protein (g)	2	2

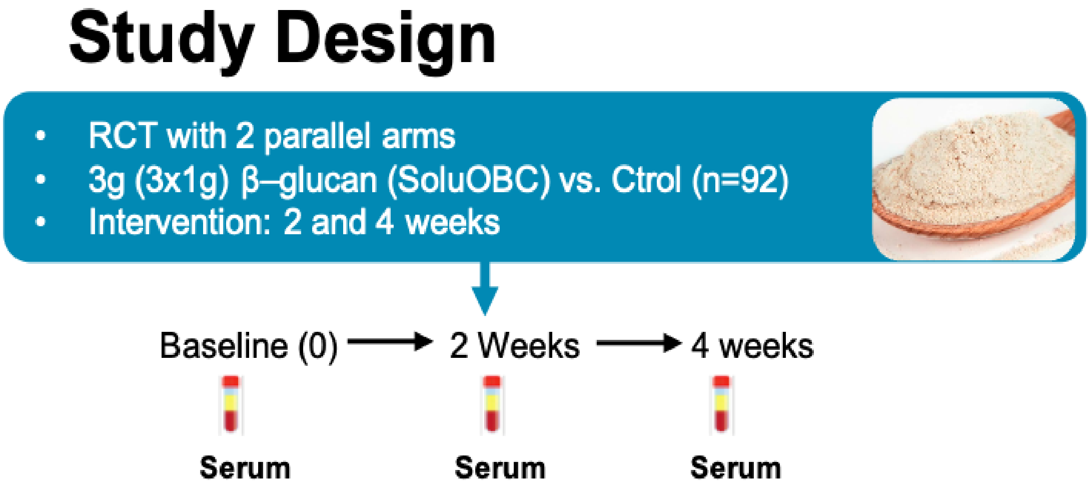

Figure S1. Study Design: randomized, double-blind, placebo-controlled, parallel-arm design. RCT, randomized controlled trial.

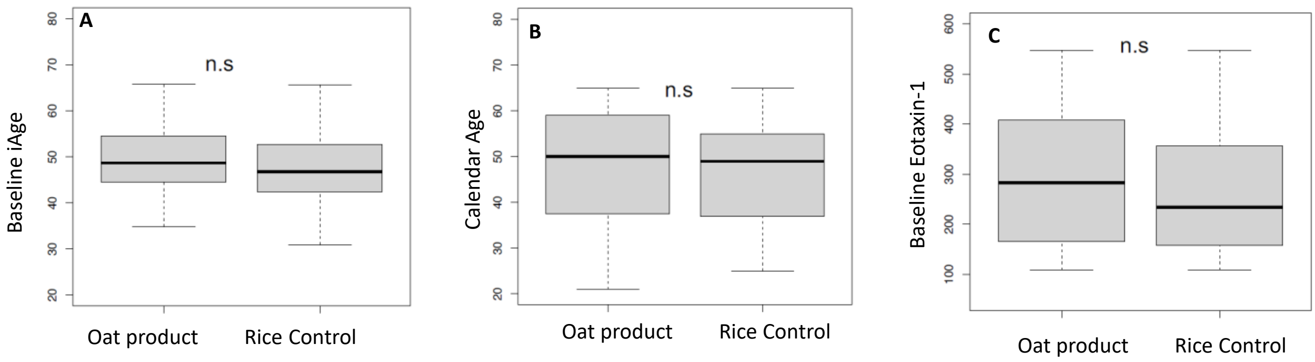

Figure S2. Description of the Study cohort at baseline. Healthy adults (n = 191) with borderline high low-density lipoprotein (LDL)-cholesterol (3 < LDL-c < 5 mM) were randomized into the oat treatment group (n = 96) or the rice control group (n = 95) with average calendar age of 48 years (range 21 to 65 years old) with 38% males and 62% female. The calculated iAge (**A**), calendar Age (**B**) and

serum Eotaxin-2 protein levels (**C**) at baseline did not significantly differ between treatment and control groups. n.s., not specified.

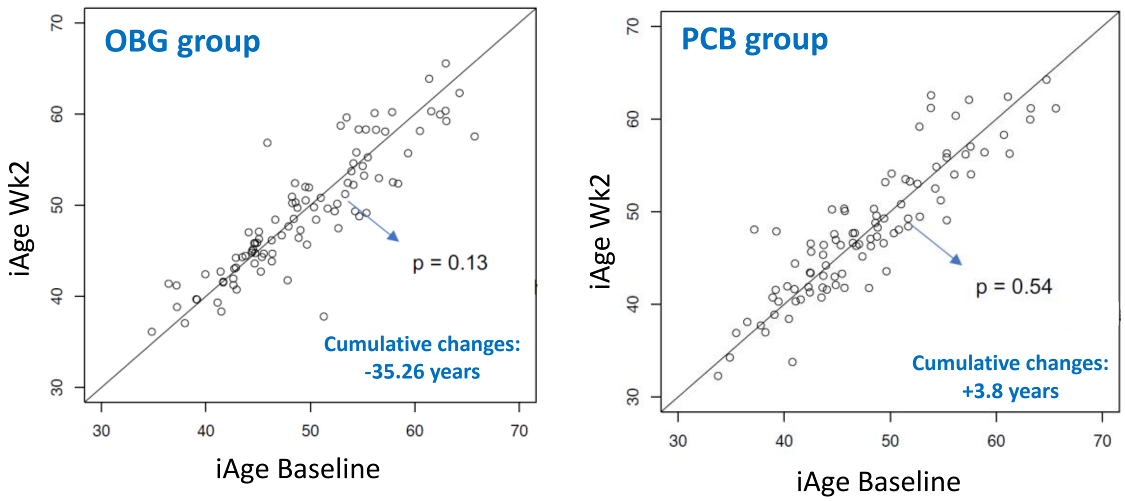

Figure S3. The Oat product (OBG) intervention group correlates with a cumulative reduction in iAge® but not the control (PCB) group. The iAge® at week 2 after intervention compared to baseline for the whole cohort, showed an overall trend, although not significant (p = 0.13) decrease of the iAge® (−35.26 years cumulatively) whereas the control PCB group showed no significant change (with a cumulative increase of the iAge® by 3.8 years).

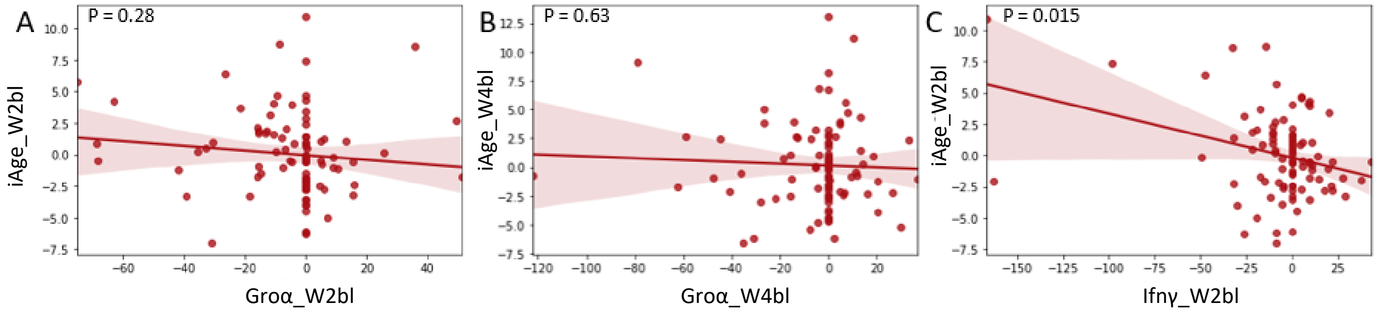

Figure S4. Changes in IFN-γand Gro-α at weeks 2 and 4 do not impact iAge. For the placebo group, Gro-α significantly decreased at weeks 2 and 4 relative to baseline, and IFN-γ significantly decreased at week 2 relative to baseline. To determine if these changes impacted SCI, the changes in these biomarkers were compared to the changes in iAge® at weeks 2 and 4. The regression statistics were obtained using scipy stats LinRegress. (**A**) Correlation between the change in Gro-α between week 2 and baseline with the change in iAge® between week 2 and baseline. (**B**) Correlation between the change in Gro-α between week 4 and baseline with the change in iAge® between week 4 and baseline. (**C**) Correlation between the change in IFN-γ, between week 2 and baseline with the change in iAge® between week 2 and baseline. Though the statistics indicate the correlation coefficient is not zero, this is largely driven by outlier data points. IFN-γ, Interferon-gamma; Gro-α, Growth Regulated Oncogene-alpha; SCI, systemic chronic inflammation.

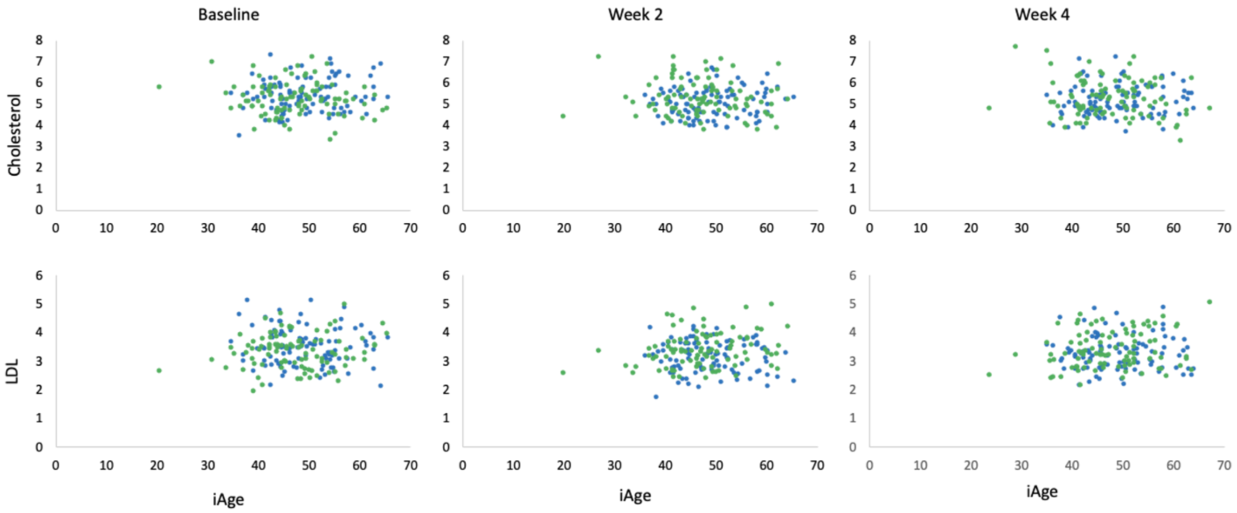

Figure S5. iAge does not correlate with HDL or LDL cholesterol levels. Each data point represents the iAge value (x-axis) compared to overall cholesterol or LDL levels (y-axis) for each patient in the oat product (blue) or placebo (green) treated groups at baseline, week 2, or week 4. HDL, high-density lipoprotein; LDL, low-density lipoprotein.
